# Puff Bars and Mental Health in Adolescents: A Danish Literature Review

**DOI:** 10.1192/j.eurpsy.2026.11152

**Published:** 2026-07-31

**Authors:** F. Monshizadeh Tehrani, C. Hansen

**Affiliations:** 1 Child and Adolescent Mental Health Servises (B102 Department of Eating Disorders); 2 Copenhagen Mental Health Center (Emergency Department), Mental Health Center in Capital Region of Denmark, Copenhagen, Denmark

## Abstract

**Introduction:**

In the past years disposable e-cigarettes (ECs) such as Puff Bars have rapidly become popular among Danish young people despite restrictions from government. High nicotine content, appealing design and flavour and easy use contribute to early exposure and popularity among adolescents. Serious consequences such as newly reported acute psychotic episode in a previously healthy adolescent after exposure (Monshizadeh & Badawey, Ugeskr Laeger 2025;1-4) raises serious concerns about potential mental health effects.

**Objectives:**

This poster aims to demonstrate the increased use of Puff Bars in Denmark, regulatory situation and associated mental health risks, focusing on potential causes for psychotic outcomes.

**Methods:**

A narrative review of national and international literature, policy reports, and case descriptions in Danish and English was conducted. The findings were combined to outline trends, risks and possible explanations for psychotic outburst.

**Results:**

In 2024 12% of Danish youth aged 15–29 years reported, three times higher in comparison with 2020. Puff Bars are a type of ECs, introduced to market in 2019. They can contain substantially higher nicotine concentrations in comparison with EU limit (up to 53 mg/ml vs 20 mg/ml). While Puff Bars have been banned in Denmark since 2024, youth have still access to them via unregulated online markets. While literature indicate the presence of toxic metals, carbonyl compounds, recent reports from public authorities raise increasing concern about synthetic cannabinoids in some products in unregulated market.

Exposure to high nicotine concentrations is associated with increased risk of various mental health concerns including dependency, anxiety and sleep disturbances. Furthermore, an association between exposure to nicotine and psychotic symptoms has also been reported. A cross-sectional study mong American students showed a link between the EC use and hallucinations and paranoia. Several explanations, including increased activity within dopaminergic pathways have been proposed. However, there is increasing evidence that contamination of ECs in unregulated market with synthetic cannabinoids can be the leading explanation.

**Conclusions:**

There is a growing concern about increasing popularity of Puff Bars among Danish youth. The early exposure to high nicotine concentration as well as chemicals from Puff Bars is associated with various mental health concerns. Recently, acute psychotic episode in a previously healthy adolescent, exposed to Puff Bars has been reported. Since 2024, its ban has pushed Puff Bars to unregulated market, where there is evidence that some products may be contaminated with substances such as synthetic cannabinoids. Aside from high nicotine concentration and may be more likely the contamination with substances can explain the development of acute psychotic episode. More research in this field is needed.

**Disclosure of Interest:**

None Declared

